# The Medical Profession

**Published:** 1843-04-01

**Authors:** 


### THE MEDICAL EXAMINER.

PHILADELPHIA, APRIL 1, 1843.

### THE PROFESSION.

By the last British Medical Journals that we have received, we find the actual condition of the profession in England depicted in no very glowing colours, and the cry for Reform, general and clamorous. The alleged causes of this state of things are the faulty system of medical legislation, and the overstocking of the profession which exists; by which means a number of unqualified persons are introdueed into its ranks, who serve by their ignorance to impair its standing in the community, and encourage the progress of quackery, which everywhere at present seems to be making rapid strides in public favour.

In England there are four distinct orders of medical practitioners—pure physicians, pure surgeons, pure apothecaries, and general practitioners. Of these, the first three are represented by corporate bodies; the last has no legal existence. There is, hence, little community of interest, and no collective influence. To remedy this, the establishment of *One Faculty* has been proposed, and stoutly advocated. The advantages of this organization are incontestable, and would doubtless ameliorate eventually the condition of the profession, raise its standard, and restore it to the confidence and respect of the public.

The multitude of practitioners—the immense disproportion between the supply and the demand—is another cause powerfully contributing to the demoralization of the profession and the success of quackery. Impatient of "hope deferred," despairing of ultimate success in a legitimate way, or impelled by want, the physician forsakes the rugged and laborious path of rectitude, and is led to adopt the smoother and more attractive one of empiricism. He apparently loses nothing in public estimation, and secures a speedy subsistence, if not a fortune.

This state of things obtains as well in our own country as abroad, and the effects of the overstocked market are as much felt in New York or New Orleans as in London or Paris, and with identical results—a desertion into the ranks of quackery.

The following article, from the Gazette Medicale, of Paris, bears so strongly on the subject that we are induced to publish a condensed translation from the Provincial Medical Journal:

"On the 9th August, 1836, a royal ordinance was published to the effect—1, that no student should be permitted to take out his first inscription unless he had the diploma of bachelor of letters;* and 2, that no student should be admitted to his first examination for the degree of doctor of medicine unless he possessed the diploma of bachelor of sciences.†

The object of this ordinance was, avowedly, to raise the respectability of the medical profession in France from the state of depression to which the concurrence of various circumstances had reduced it.

---

* For this diploma the student is examined during three-quarters of an hour in the Greek, Latin, and French languages; philosophy, ancient and modern history, and ancient and modern geography.

† For this diploma the student is examined in mathematics, physics, chemistry, zoology, botany, and mineralogy.

This depressed state was chiefly indicated by an evident diminution of *esprit de corps* in the medical commonwealth; by excessive competition amongst medical men; the influx of charlatans; relaxation of professional morality; and, finally, by the wretched condition of the art of medicine as a means of existence for its professors.

Such clear symptoms of moral and material decay could not fail to awaken the attention of all well-thinking men. Various plans of medical reform were concocted; the Chamber of Deputies was beset by petitions; severe measures of repression were demanded against quacks and backsliding professionals; and 'benevolent funds' were instituted for the succour of destitute doctors. The various plans thus imagined for the relief of the profession seemed excellent upon paper; but the difficulty lay in their execution. A law was promised, but that law still slumbers in the portfolio of the minister, and may, perhaps, never see the light.

The evils under which the profession laboured were, however, too pressing to wait the expectant treatment of the minister, and an attempt was made to remedy them indirectly by the ordinance already alluded to. The objects of this regulation were the same as those invoked by all classes of medical reformers, but its chief results were twofold. It tended in the first place to diminish the number of medical men; and in the second, to increase the respectability of the profession, by augmenting the amount of scientific instruction and the proofs of capability in each individual member of that profession.

This twofold object being attained, it was clear that most of the moral and material evils which beset the profession would be greatly alleviated, if not radically removed. The event has justified the prudent foresight which dictated the ordinance. The radical source of the abuses complained of—viz., the shameful increase of quackery, the degraded condition of the medical profession, and its utter worthlessness as a means of honorable livelihood; all these clearly depended on an over supply of medical practitioners. The instant that the supply considerably exceeded the demand, it followed, as a matter of necessity, that vast numbers of medical men were driven from the legitimate means of existence. The field, previously divided between them in a fair and peaceful manner, now became the scene of hot and desperate contest. The character of the profession was at once degraded, and a war of interests was substituted for an honorable competition of talent. Medicine was no longer regarded as a means of quiet, though dignified support; a few scandalous examples blinded men's eyes, and the science degenerated into the trade. The bonds of brotherhood being thus rent asunder, the commonwealth of medicine lost its character of fraternity, and became a battle-field where every man's hand was raised against his neighbour. Individual success was regarded as the sole test of merit, and the acquirement of wealth the sole object of existence. The effects of such a state of things were inevitable. Science, the cultivation of the understanding, and literature, were regarded as mere accessories; the student contented himself with the amount of technical knowledge requisite to pass his examination, and the noblest of professions sunk into the most degrading of trades. This picture may be somewhat highly colored, but the representation is faithful.

The over-crowding of the profession is evidently the source of the chief evils under which we labour; if this be admitted, the remedy is clearly indicated. The supply of medical practitioners must be reduced within limits somewhat commensurate with the demand for them. In a country like France no one would listen to any proposal for limiting the number of practitioners by legislative enactment; this is constitutionally impossible; but the same result may be attained indirectly by raising the standard of medical education, and thus cutting off the excess of candidates who have been attracted by the facility of entering the profession. Such, we repeat, was the object of the ordinance of 1836, which has completely realised the expectations founded on it. The following table illustrates the practical working of this regulation:—

| Years. | University Pupils. | Pupils at Prov. Schools. | Total. |
|---|---|---|---|
| 1835 | 1095 | 427 | 1522 |
| 1836 | 750 | 340 | 1090 |
| 1837 | 458 | 286 | 744 |
| 1838 | 295 | 301 | 596 |
| 1839 } 1840 } 1841 } | Nearly as in 1838. | | |
| 1842 | 321 | 307 | 628 |

From the preceding table, it appears that the effect of increasing the standard of preliminary education was at once to reduce the number of medical students from 1522 to 1090. This diminution continued during the years 1837 and 1838; and the number then continued stationary, having arrived at what may fairly be regarded as the normal standard. During last year the number of new students admitted at Paris was 200; at Montpellier, 98; at Strasbourg, 23. In the preparatory schools of the provinces the relative diminution was by no means so great."

